# Polyimide Aerogels Cross-Linked with Aminated Ag Nanowires: Mechanically Strong and Tough

**DOI:** 10.3390/polym9100530

**Published:** 2017-10-19

**Authors:** Tianyi Zhang, Yan Zhao, Kai Wang

**Affiliations:** 1 School of Material Science & Engineering, Beihang University, Beijing 100191, China; wing52711@126.com (T.Z.); jennyzhaoyan@buaa.edu.cn (Y.Z.); 2 Key Laboratory of Aerospace Materials and Performance, School of Material Science & Engineering, Beihang University, Beijing 100191, China

**Keywords:** polyimide aerogels, Ag nanowires, amino functionalization, nanoparticle cross-linker, mechanical properties

## Abstract

In this study, polyimide (PI)/Ag nanowire (AgNW) nanocomposite aerogels with extremely high mechanical performance have been fabricated utilizing amine-modified AgNWs as mechanical nanoreinforcement particulates and crosslinking agents. Initially, AgNWs were fabricated and surface modified by *p*-aminothiophenol (PATP), then the aminated AgNWs were dispersed into polyamide acid solution and aerogels were prepared by supercritical CO_2_ drying. Raman and X-ray photoelectron spectroscopy (XPS) spectrometry were carried out on A-AgNWs (aminated Ag nanowires) to prove the successful modification. This functional nanoparticle greatly enhanced the strength and toughness of aerogels without evident increase in densities. Comparing to pure PI aerogels, samples with 2.0 wt % of A-AgNWs had a 148% increase in compression strength and 223% increase in Young’s modulus, which equates to 2.41 and 27.66 MPa, respectively. Simultaneously, the tensile test indicated that aerogels with 2.0 wt % of A-AgNWs had a breaking energy of 40.18 J/m^3^, which is 112% higher than pure PI aerogels. The results presented herein demonstrate that aminated AgNWs are an innovative cross-linker for PI aerogels and can improve their strength and toughness. These aerogels have excellent potential as high-duty, lightweight porous materials in many areas of application.

## 1. Introduction

As one of the most popular organic aerogels, polyimide (PI) aerogels have attracted considerable attention in recent years. Comparing to the conventional silica aerogels [1,2], PI aerogel not only possesses the typical properties of aerogels [3], such as low density, high porosity, good insulativity and small pore size [4], but also shows the excellent advantage of flexibility [5], combined with outstanding thermostability and fire resistance [5] unlike other conventional polymer aerogels [6], such as syndiotactic polystyrene [7], polyurethane [8,9,10], polyurea, cellulose [11], poly(vinyl alcohol) [12], polyamide [13,14], chitin [15] etc. As a consequence, PI aerogels have been intensively investigated due to their wide range of potential applications in aerospace [16] and construction industries [17,18] as thermal and electric insulation materials [19].

There have been some pioneering studies on PI aerogel [20]. From NASA (National Aeronautics and Space Administration), Meador et al. [21,22,23] used 1,3,5-triaminophenoxybenzene (TAB) and 1,3,5-benzenetricarbonyl trichloride (BTC) as cross-linkers to prepare a series of PI aerogels with different dianhydride and diamine by supercritical CO_2_ drying. The results showed that the density of the PI aerogel could be as low as 0.14 g/cm^3^ and the specific surface area could be as high as 512 m^2^/g. Besides, the glass temperature could be improved to 340 °C. However, the mechanical properties of these PI aerogels were not remarkable. Guo et al. [24,25] prepared PI aerogel with 3,3’,4,4’-biphenyltetracarboxylic dianhydride (BPDA) and bisaniline-pxylidene (BAX) using a cross-linker octa-(aminophenyl) silsesquioxane (OAPS). The produced PI aerogel had smaller shrinkage due to the eight crosslinking sites provided by OAPS, and could reduce the density to 0.1 g/cm^3^; nevertheless, with a decrease in special surface area to 260 m^2^/g and an unimproved compression module of 1.7~5.3 MPa. Leventis [26] prepared PI aerogel monoliths via ring-opening metathesis polymerization (ROMP) of a norbornene end-capped diimide, bis-NAD [2,2′-(methylenebis(4,1-phenylene))bis(3a,4,7,7a-tetrahydro-1H-4,7-methanoisoindole-1,3(2H)-dione)]. Young’s modulus of the products reached a high level of about 288 MPa, yet the bulk densities were over 0.6 g/cm^3^, which is not ideal for light-weight applications.

In the aforementioned studies, cross-linkers are mainly small molecules, including TAB, OAPS, and tri(3-aminophenyl)phosphine oxide (TAPO) [27], which are very complicated to synthesize and are generally expensive. Instead of using small molecule cross-linkers, adding nanoparticles into aerogels appears to be a reliable method for their modification. Nanoparticles such as graphene oxide [28], nano-silica, carbon nanotubes [29,30,31], MOFs (Metal Organic Frameworks) [32] and cellulose nano-fibers [33] are showing promise to act as physical cross-linking points and reinforcement at the same time, which may improve the properties of aerogels remarkably.

Among various kinds of nanoparticles, Ag nanowires (AgNWs) are a kind of one-dimensional nano-material with excellent properties. In our previous work [34,35,36], controllable high-concentration AgNWs have been synthesized rapidly on a large scale by a facile and effective two-step dropping polyol method. Due to their high aspect ratio and great mechanical properties, AgNWs are considered to be ideal reinforcement in the polymer matrix. Furthermore, if treated with amino-functionalization, AgNWs can provide a great number of chemical cross-linking points and serve as reinforcement simultaneously, which should enhance the mechanical properties of aerogels to a great extent.

Hence, in this study, we prepared AgNWs with an aspect ratio of 30 followed by amination modification via the introduction of *p*-aminothiophenol (PATP), as PATP has been proven to be an excellent modifier for nano-silver [37,38]. As a cross-linker, aminated Ag nanowires (A-AgNWs) were then added into 10% *w*/*w* % poly (amic acid) (PAA) oligomers solution end-capped by anhydrides and finally prepared into polyimide aerogels. As the results indicate, PATP has been successfully grafted on AgNWs by chemical bonding; the final products have a slight increase in densities but significantly rise in strength and toughness. Specifically, when contents of A-AgNWs in aerogels increased from 0 to 2.0 wt %, their densities rose from 0.192 to 0.205 g/cm^3^. However, samples with 2.0 wt % of A-AgNWs had a 148% increase in compression strength and a 223% increase in Young’s modulus compared to pure PI aerogel samples, which equates to 2.41 and 27.66 MPa, respectively. Simultaneously, the tensile test indicated that aerogels with 2.0 wt % of A-AgNWs had a breaking energy of 40.18 J/m^3^, which is 112% higher than pure PI aerogels. Thus, this type of relatively high-duty aerogels has great potential in applications of absorption, construction and aerospace as lightweight porous materials. Furthermore, if the mass fraction of A-AgNWs in polymer increases to a higher level, the aerogel could find numerous applications such as catalysis, EMI (Electronic-Magnetic Interference) shielding, etc.

## 2. Materials and Methods

### 2.1. Materials

AgNO_3_, NaCl, Dimethylacetamide (DMAc), PATP, triethylamine and ethanol were all supplied by Beijing Finechem, Beijing, China. Ethylene glycol (EG) and polyvinylpyrolidone (PVP, *M*_w_ ≈ 40,000) were purchased from Xilong Chemical Industry Incorporated Co., Ltd., Shantou, China. BPDA was purchased from Pome Sci-tech. Co., Ltd, Beijing, China and was dried in a vacuum oven at 180 °C for 8~10 h prior to use. The 4, 4′-Diaminodiphenyl ether (ODA) and acetic anhydride were obtained from Sinopharm Chemical Reagent Co., Ltd., Shanghai, China. All reagents were analytical grade.

### 2.2. Preparation of A-AgNWs

**Figure 1:**
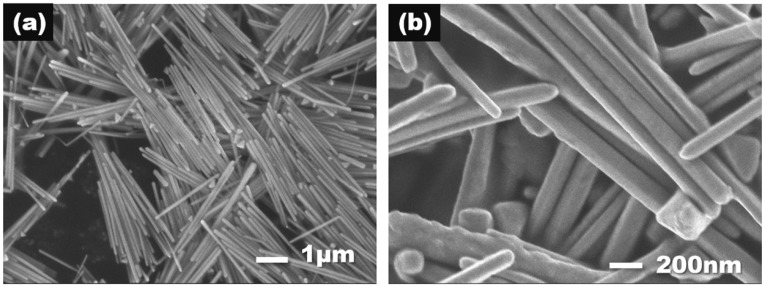
SEM images of (**a**) Ag nanowires (AgNWs) and (**b**) aminated Ag nanowires (A-AgNWs).

AgNWs were synthesized by the two-step dropping polyol process, as previously reported by our group [36]. In this typical synthesis procedure, 40 mL of 0.56 M PVP solution in EG was heated to 160 °C until the temperature was steady. Afterward, 100 μL of 0.15 M NaCl solution in EG was injected into the mixture above. Then, 10 mL of 1.5 M AgNO_3_ solution in EG was added into the dropping funnel and then dropped into the flask at the rate of 15 μL/s at first. Once the reaction solution turned from darkorchid to celadon/greyish-green, which indicated the presence of silver nanoparticles and pentagonal twinned decahedron particles, all the remaining AgNO_3_ solution was added into the flask immediately. After 2 h, the reaction was quenched and the suspension was diluted with ethanol (in a weight ratio of 1:10) and centrifuged five times at 6000 rpm for 20 min. The washed products were dried in a vacuum oven at 60 °C for 1 h and stored at below room temperature for further amination. The average diameter and length of the nanowires were found to be 100 nm and 6 μm (seen Figure 1), respectively.

The aforementioned AgNWs were weighed and re-dispersed in ethanol, then executed with ultrasonic treatment using a bath sonicator for 60 min in order to achieve optimum uniform dispersion. Subsequently, PATP was added into the system in a molar ratio of 1:4. This mixture was magnetically stirred for 24 h at room temperature, afterward centrifuged and ultrasonic dispersed alternately four times till the supernatant liquid turned colorless and transparent. The final products, noted as A-AgNWs, were dried and stored following the same method as with AgNWs.

### 2.3. Preparation of A-AgNWs Cross-Linked Polyimide Aerogel

**Scheme 1:**
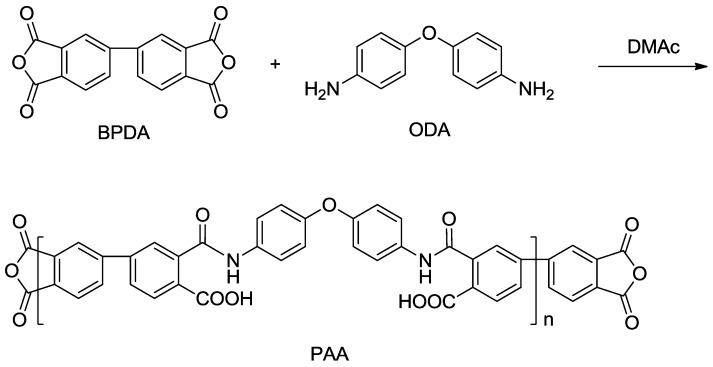
Synthesis of Polyamide Acid (PAA) prepolymer.

Polyamide acid prepolymer was synthesized as Scheme 1, where DMAc served as non-aqueous polar solvent. A formulated ratio of (*n* + 1) BPDA to *n* ODA was used in order to control the molecular weight of polymers which contain excess anhydrides for cross-linking. The solid content of polyamide acid solution was set at 10 wt %. Meanwhile, A-AgNWs were homogeneously dispersed into the polyamide acid solution as cross-linkers. The mass fraction of A-AgNWs was designed to be 0%, 0.1%, 0.2%, 0.5% and 2.0% respectively relative to polyamide acid. Notably, the maximum content of A-AgNWs was set at 2.0% since higher content may cause undesirable conglomeration of particles. This phenomenon was proved in experiments. The procedure for preparing polyamide acid solution whose degree of polymerization was 50 was as follows: BPDA (4.501 g, 15.3 mmol) in 42.55 g DMAc was added into the solution with ODA (3.005 g, 15 mmol) and 20 g DMAc. During the preparation, BPDA should be considerably slowly added to avoid overheating which may lead to implosion; besides, an ice-water bath was recommended when the environment temperature is above 25 °C. The solution was stirred for 24 h. Afterwards, A-AgNWs at a designed weight was added and reacted for another one hour. The polyamide acid was then imidized by triethylamine (4.554 g, 45 mmol, 3:1 molar ratio to BPDA) and acetic anhydride (4.593 g, 45 mmol). The solution was continually stirred for 5 min and then poured into 5 ml centrifuge tubes and 140 mm × 70 mm × 20 mm boxes to obtain cylindrical and sheet samples, respectively.

Gels were aged in a mold for 24 h and then soaked into DMAc for another 24 h to remove the triethylamine and acetic anhydride. Afterwards, solvent exchange was performed by replacing the solution with ethyl alcohol every 12 h. This procedure lasts for 48 h before it is accomplished. Afterwards, those gels were submerged in ethyl alcohol in a sealed chamber filled with liquid CO_2_ at 14 MPa and 25 °C, and CO_2_ was exchanged every 2 h until hardly any ethyl alcohol remained in the chamber; the exchange generally takes 24 h. Then, the temperature of chamber was set to 45 °C and the pressure was decreased to 12 MPa to turn CO_2_ into supercritical state and hold for 5 h followed by a slow venting for nearly 8 h. The obtained products were aged at 80 °C for 12 h in a vacuum oven to ensure a complete imidization. The final products were numbered as PI-wt %( A-AgNWs). Notably, all samples should be made from one same portion of polyamide acid prepolymer in order to control every variable except for cross-linker contents.

### 2.4. Characterization

A JEOL FESEM JSM 7500 (JEOL Ltd., Tokyo, Japan) was used to investigate the microstructure of AgNWs as well as the aerogels, with the latter samples coated with platinum. Raman spectrometry of AgNWs and A-AgNWs was performed on LabRAM HR800 produced by HORIBA Jobin Yvon Instrument Co., Ltd., Paris, France. XPS spectra were obtained by ESCALAB 250Xi, Thermo Fisher Scientific, Waltham, MA, USA. BET (Brunauer–Emmet–Teller) specific surface area was measured by the nitrogen sorption method using a 3H-2000PS2 (Beishide, Ltd., Beijing, China) analyzer; the samples were outgassed at 80 °C for 12 h before testing. The apparent density (ρ) of aerogels was calculated by measuring the weight and the volume of the samples. Compression and tensile tests were performed on a HY-0350X universal material testing machine made by Shanghai HengYi Precision Instrument Co., Ltd., Shanghai, China. The specimens for compression were tested between a pair of compression platens with GB T8813-2008. Simultaneously, the tensile test was carried out according to ASTM D638 2003, where the thickness of samples was adjusted from 4 to 2 mm. Reported mechanical properties are the average of at least five tests.

## 3. Results and Discussion

### 3.1. Characterization of A-AgNWs

The morphologies of the AgNWs and A-AgNWs were examined using field emission scanning electron microscopy (SEM). Images of both AgNWs and A-AgNWs are shown in Figure 1. The average length and diameter of AgNWs and A-AgNWs can be measured as 4 μm and 150 nm, respectively, which means they possess a high aspect ratio of 30. Surface appearance showed hardly any change after the modification, therefore Raman spectromentry and XPS are required to further verify the existence of PATP on the surface of A-AgNWs as well as their binding state.

**Figure 2:**
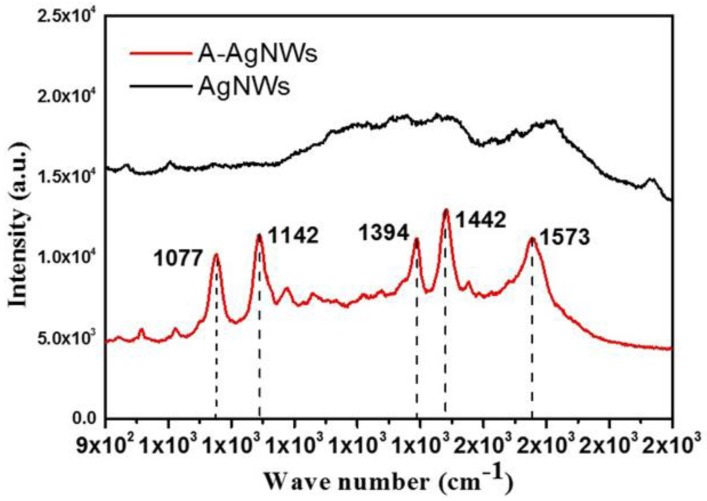
Raman spectra of AgNWs and A-AgNWs.

Raman spectra of AgNWs and A-AgNWs are shown in Figure 2. From the result, it can be seen that the spectra of two substances visibly varied from each other. The spectrum of AgNWs obtained indistinct peaks at around 1390, 1440 and 1580 cm^−1^, which correspond to the stretching vibration of the C–N, and C–C bonds and in-phase vibration of the C–C bond that exist in PVP absorbed on the surface of AgNWs. After the amination, these aforementioned peaks become more intense, suggesting that the introduction of PATP brought more C–N and C–C bonds. In addition, new peaks at 1077 and 1142 cm^−1^ can be clearly observed, which correspond to the a_1_ vibrational modes (in-plane, in-phase modes) of the C–S bond and b_2_ modes (in-plane, out-of-phase modes) of the C–C bond, respectively. The former result could be further proof of the existence of PATP, while the latter can be ascribed to the charge transfer of the metal to the adsorbed molecules and demonstrates that PATP in these cases adopts a perpendicular orientation to the metal surface, which means that PATP is attached on the smooth silver surface with its sulfur atom by forming a chemical bond.

**Figure 3:**
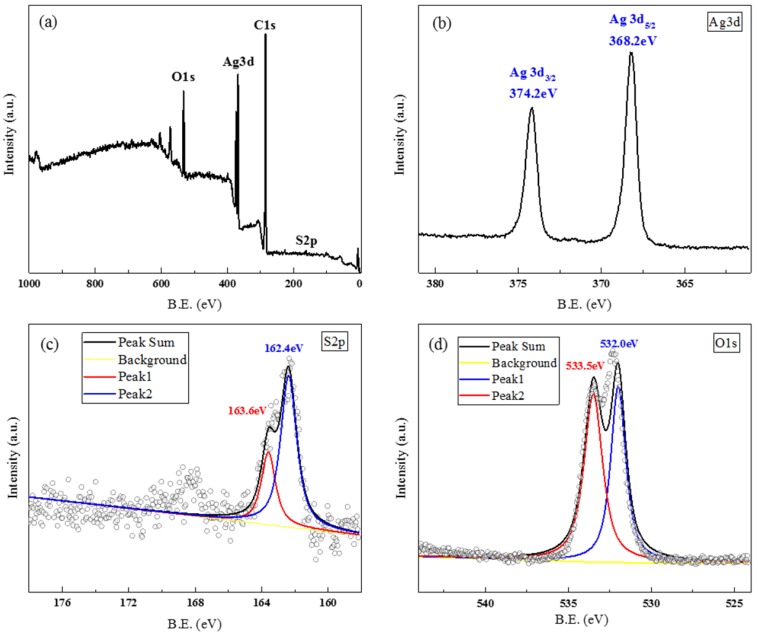
XPS spectra of A-AgNWs: (**a**) survey; (**b**) Ag 3d; (**c**) S 2p; and (**d**) O 1s high resolution spectrum.

Figure 3a–d show the high resolution XPS spectra of A-AgNWs. Calibration was processed according to the C1s spectrum, whereupon the peaks in other spectra were ensured to be accurate. The spectrum of Ag 3d contained two peaks; the peak at 374.2 corresponds to 3d_3/2_ peaks of metallic silver, meanwhile the 3d_5/2_ peaks of metallic silver and Ag bonded to sulfydryl are both around 368.2 eV. Furthermore, in the results of the S 2p scan, two peaks at 162.4 and 163.6 eV respectively, were obtained after optimizing. According to statistics in the NIST XPS database, those peaks represent the binding energy of sulfydryl in PATP to Ag. Those two spectra represent strong evidence that PATP has successfully connected to AgNWs by chemical bonding. Besides, in the O 1s spectrum, the carbonyl group of PVP was detected at 532.0 and 533.5 eV, which indicates that the surfaces of A-AgNWs are strongly coordinated with O atoms in PVP. Thus, the coordination interaction of PVP on the surface of A-AgNWs will promote effective interactions between A-AgNWs and the polymer. Both of these observations prove that adding PATP to Ag nanowires appears to be a feasible method for its surface modification.

### 3.2. Properties of A-AgNWs Cross-Linked Polyimide Aerogels

**Figure 4:**
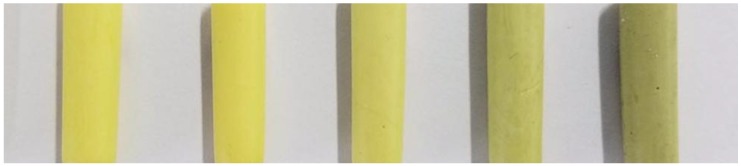
Appearances of polyimide (PI) aerogels with different A-AgNWs contents. From left to right PI-0, PI-0.1, PI-0.2, PI-0.5, PI-2.

Polyimide aerogels cross-linked by A-AgNWs were obtained via the aforementioned methods. As shown in Figure 4, the produced aerogels are basically yellow in appearance; from left to right, the contents of A-AgNWs are 0%, 0.1%, 0.2%, 0.5% and 2.0%. The colors of aerogels differ from citrine to dark khaki, that is, the more A-AgNWs the aerogel contains, the greener it appears.

Shrinkage occurs during fabrication of the aerogels, mostly during initial gelation but some additional shrinkage may occur during the solvent exchange and supercritical drying. Stats in Table 1 shows that with the increase of A-AgNWs content, the density of PI aerogels slightly augmented from 0.192 to 0.205 g/cm^3^, which is rational due to the density of silver reaching 10.9 g/cm^3^. However, shrinkage during the drying and the imidization reaction led to a reduction of 13%, with PI-0 a reduction of 16.5% and with PI-2 a reduction of 14.3%. As can be seen, the crescent volume fraction of A-AgNWs in PI aerogels increased the occurrence of cross-linking reaction, which makes A-AgNWs rigid backbones for supporting molecular chains in aerogels during supercritical CO_2_ drying as well as the imidization reaction, thus keeping aerogels from shrinking. That may explain the decrease in shrinkage. The surface area measurements were made by nitrogen sorption using the Brunauer–Emmet–Teller (BET) method, as shown in the table. The surface areas of different groups of aerogels were around 260 m^2^/g, which did not significantly vary with the increase of A-AgNWs contents.

**Figure 5:**
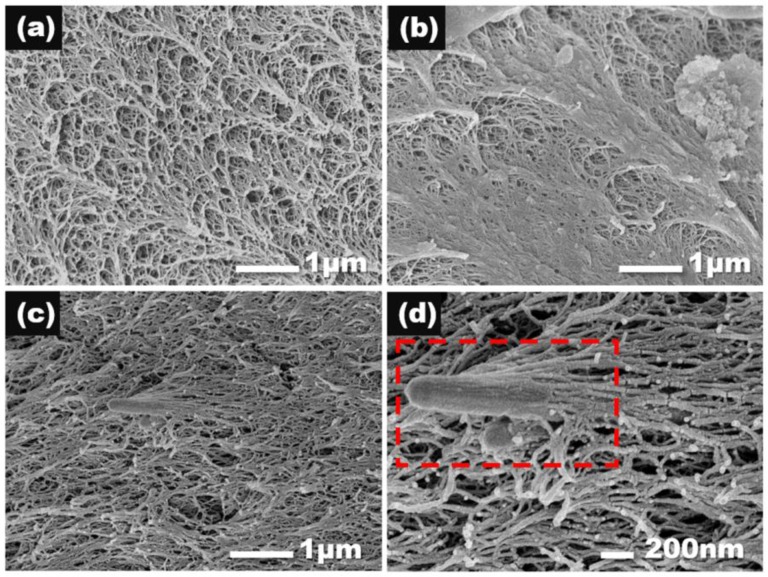
SEM images of aerogel samples. (**a**) PI-0 treated with liquid nitrogen; (**b**) PI-0 for tensile test; (**c**) PI-2; (**d**) PI-2 in high resolution.

The morphologies of the aerogel monoliths were examined using SEM as shown in Figure 5. Notably, samples in Figure 5a,b had identical chemical constitutions, but were treated differently when made into SEM samples. Specifically, Figure 5a was soaked in liquid nitrogen to achieve brittle facture, while Figure 5b broke due to tensile failure. Among SEM images of aerogels with different A-AgNWs, aerogels of different A-AgNW contents had a similar porous microstructure, in accordance with the results of densities, shrinkages and surface areas, which could demonstrate that the introduction of A-AgNWs will not affect the micromorphology of PI aerogels. Comparing Figure 5a with Figure 5b, it can be found that the appearance of a fracture was due to the combination of porous structure and a flat structure, as the flat structure can be deemed to be a deformation from the porous structure while being torn and stretched during tensile fracture.

Notably, when A-AgNWs were relatively less contained, it is hard to locate their exact position in SEM images. However, as shown in Figure 5c, A-AgNWs were found in PI-2 samples, where the volume fraction of A-AgNWs reached 0.27%. At a higher magnification, as shown in Figure 5d, it is evident that A-AgNWs were attached to the polyimide matrix in the same way that a tree trunk is covered with branches. Some polyimide molecule strands were grafted to A-AgNWs on one side and connected to other molecule strands in aerogel network structures on the other side. This was direct evidence that A-AgNWs were acting as cross-linking nodes. Furthermore, under the easily noticeable half-exposed A-AgNWs, a front end of another A-AgNWs could be found in Figure 5d, demonstrating that the latter was almost completely buried in the polyimide matrix. From these appearances in Figure 5d, it is reasonable to assume that A-AgNWs were orientated disorderly in aerogels, as demonstrated in the graphic abstract. This would provide aerogels with good mechanical properties isotropically.

**Figure 6:**
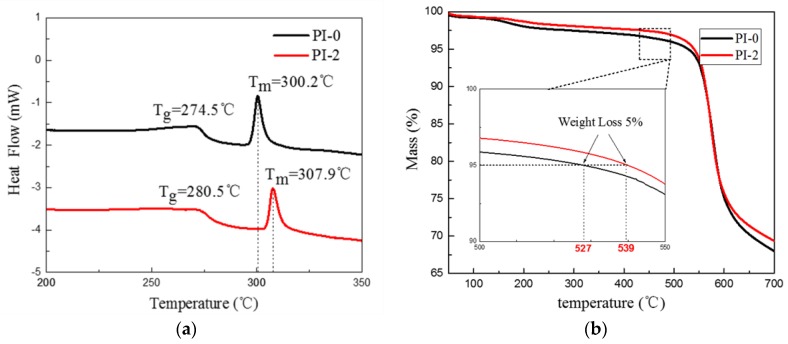
(**a**) DSC curves of PI-0 and PI-2; (**b**) TGA curves of PI-0 and PI-2.

DSC(differential scanning calorimetry) and TGA(thermogravimetric analysis) of PI-0 and PI-2 were performed to study the effect of the addition of A-AgNWs on the thermal performance of aerogels. A DSC test was performed at a temperature range of 25 to 400 °C with a heating rate of 10 °C/min. From the DSC results in Figure 6a, it is shown that the curves of both samples corresponded to the typical DSC curve of BPDA-derived PI, i.e., both curves had a glass transition platform and an endothermic melting peak, and main variations occurred between 200 and 350 °C. Comparing to PI-0, the addition of 2.0% wt % of A-AgNWs in PI-2 led to the *T*_g_ of PI aerogel rising from 274.5 °C to 280.5 °C, along with its *T*_m_ rising from 300.2 to 307.9 °C. Simultaneously, TGA was tested from room temperature to 800 °C on both samples, with a heating rate of 10 °C/min. Shown in Figure 6b, as the TGA results present, main weight loss occurred after 500 °C. Comparing to PI-0, temperature at 5% weight loss of PI-2 goes up from 527 to 536 °C, i.e., the incorporation of A-AgNWs led to a slight improvement in the thermal performance of PI aerogels.

**Figure 7:**
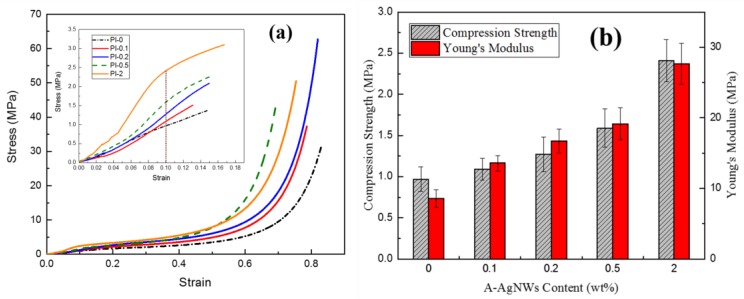
Results of compression tests: (**a**) stress–strain curves of PI-0 to PI-2; (**b**) statistics of compression strength and Young’s modulus of PI-0 to PI-2.

Except for measuring the densities and shrinkages of the aerogels, the effect of A-AgNWs on the mechanical properties of aerogels was further studied through compression and tensile tests. Figure 7a,b show the results of compression tests for PI aerogels. From the shape of the stress–strain curve on its yield fragment, it is obvious that all the samples presented good strength and toughness. Normally, BPDA-derived PI aerogels had better toughness than PMDA-derived PI, and a higher modulus than PI aerogels which had BTDA as dianhydride. Additionally, in this study, polyimide with its *n* = 50 was synthesized, which was normally under 40. This level of high polymerization would improve the mechanical properties of aerogels to some degree.

However, the addition of A-AgNWs was proven to lead to evident growth in strength and the Young’s modulus. As shown in Figure 7b, comparing with PI-0 which processes a compression strength of 0.97 MPa, PI-0.1 showed 12% growth at 1.09 MPa, which rose by 12.4%. Furthermore, when A-AgNW content was added up to 2.0 wt %, the compression strength of aerogels significantly increased to 2.41 MPa which improved by 148% with respect to aerogels with no A-AgNWs. Young’s modulus stats show a similar pattern to aerogels with 2.0% A-AgNW’s own modulus of 27.66 MPa, which was 223% more than Young’s modulus of blank samples. AgNWs endowed great improvement in strength, homogenously dispersed among aerogels, which was quite convictive according to the test results.

**Figure 8:**
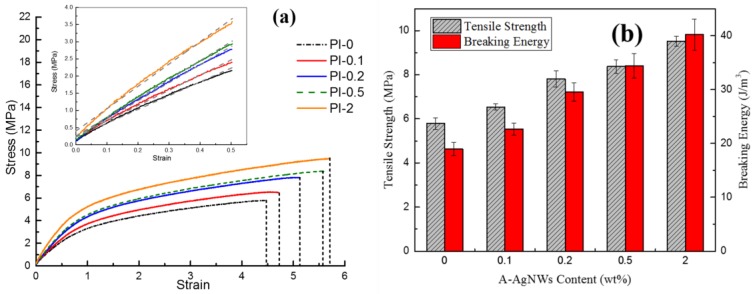
Results of tensile tests (**a**) stress–strain curves of PI-0 to PI-2; (**b**) statistics of tensile strength and breaking energy of PI-0 to PI-2.

Figure 8a,b show the results of tensile tests for aerogels; with the increasing addition of A-AgNWs, tensile strength and modulus showed evident growth, similar to the compression experiment. Comparing with PI-0 which had a strength of 5.79 MPa, PI-0.1 showed 13% growth at 6.54 MPa. When A-AgNW content was added up to 2.0%, the tensile strength reached 9.52 MPa which was a 64% increase compared to the control sample. In addition, the elongation at the break showed a relatively remarkable increase as shown in the curves; by integrating each curve, the breaking energies of every sample are shown in Figure 8b. The breaking energy results indicated that the presence of A-AgNWs could significantly improve the strength and toughness of aerogels at the same time, which is rarely achieved.

**Figure 9:**
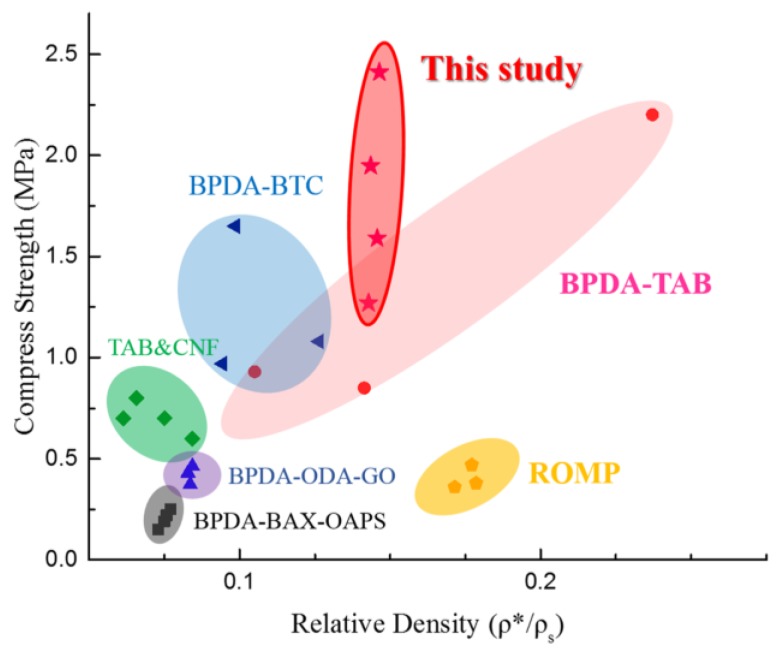
Comparison of aerogels’ compression strength in this study compared to others’ previous work.

The reason why A-AgNWs greatly improved both the strength and toughness of aerogels simultaneously may be seen from two aspects. On the one hand, AgNWs orientated randomly in polyimide aerogels; these two constituents formed metal-enhanced polymer composite, where silver nanowires served as tough and rigid metallic reinforcement to enhance the strength of the polyimide matrix. This is highly consistent with the results of compression tests. On the other hand, owing to its high aspect ratio and surface area, AgNWs possessed a lot of cross-linking points after being modified with PATP, which leads to a sufficient cross-linking reaction between A-AgNWs and polyimide. In that case, with the increase of A-AgNW contents, the cross-linking density increased significantly, which led to great improvement in the mechanical properties of PI aerogels, especially with regard to the toughness of aerogels. We compared our mechanical properties results with several authoritative studies on PI aerogels, as shown in Figure 9. The samples in this study show comparatively high compression strength with similar densities.

## 4. Conclusions

Polyimide aerogels cross-linked with aminated Ag nanowires (A-AgNWs) were prepared by surface functionalization along with supercritical CO_2_ drying. Ag nanowires were synthesized by the two-step dropping polyol process, after washing and drying, modification was performed on AgNWs with *p*-aminothiophenol (PATP). The results of Raman spectroscopy and XPS combined to prove that PATP successfully grafted to AgNWs by chemical bonding.

BPDA-ODA-based polyimide with polymerization *n* = 50 was synthesized at room temperature, then the total solids composition of the wet gels was kept constant at 10 wt %, with 0~2.0 wt % of the total solids being A-AgNWs. Samples of different A-AgNW content were characterized via SEM, BET, DSC, TGA and a mechanical test. With the increase in A-AgNW contents, densities slightly augmented from 0.192 to 0.205 g/cm^3^; shrinkage during drying and the imidization reaction led to a reduction of 13%, and PI-2 only shrank 14.3%. It was observed in images that A-AgNWs were covered with polyimide molecule strands, which proved that the aminated silver nanowire effectively embody the bonding to polyimide. Besides, from the DSC and TGA results, the incorporation of A-AgNWs led to a slight improvement in the thermal performance of PI aerogels.

Most notably, as shown in the results of mechanical tests, A-AgNWs led to a great enhancement in the strength and toughness of aerogels at the same time. Specifically, the compression strength rose from 0.97 to 2.41 MPa, Young’s modulus increased by 223%, and breaking energy increased two-fold compared to the blank sample. These results suggest that aminated AgNWs are an advisable cross-linker for PI aerogels, which have great potential in applications of absorption, construction and aerospace as lightweight, high-duty and thermostable porous materials.

## Figures and Tables

**Table 1:** Formulation and properties of aerogels with different A-AgNWs contents.

Run	Polyimide conc. (wt %)	A-AgNWs conc. (wt %)	AgNWs conc. (vol %)	Density (g/cm^3^)	Shrinkage (%)	Surface area (m^2^/g)
1	10	0	0	0.192	16.5	256
2	10	0.1	0.013	0.191	15.8	266
3	10	0.2	0.027	0.200	16.0	254
4	10	0.5	0.067	0.204	14.8	257
5	10	2.0	0.270	0.205	14.3	248
